# Anti-Obesity Effects of Granulocyte-Colony Stimulating Factor in Otsuka-Long-Evans-Tokushima Fatty Rats

**DOI:** 10.1371/journal.pone.0105603

**Published:** 2014-08-21

**Authors:** Yonggu Lee, Yi-Sun Song, Cheng-Hu Fang, Byung-Im So, Jun-Young Park, Hyun-Woo Joo, In-Hwa Park, Guang-Yin Shen, Jeong-Hun Shin, Hyuck Kim, You-Heon Ahn, Kyung-Soo Kim

**Affiliations:** 1 Division of Cardiology, Department of Internal Medicine, Hanyang University College of Medicine, Seoul, South Korea; 2 Graduate School of Biomedical Science and Engineering, Hanyang University, Seoul, South Korea; 3 Division of Cardiology, Yanbian University, Yanji, China; 4 Department of Thoracic Surgery, Hanyang University Hospital, Seoul, South Korea; 5 Department of Endocrinology, Hanyang University Hospital, Seoul, South Korea; 6 Department of Cardiology, Sung-Ae Hospital, Seoul, South Korea; Institut National de la Santé et de la Recherche Médicale, France

## Abstract

Granulocyte-colony stimulating factor (G-CSF) has molecular structures and intracellular signaling pathways that are similar to those of leptin and ciliary neurotropic factor (CNTF). It also has immune-modulatory properties. Given that leptin and CNTF play important roles in energy homeostasis and that obesity is an inflammatory condition in adipose tissue, we hypothesized that G-CSF could also play a role in energy homeostasis. We treated 12 38-week-old male Otsuka-Long-Evans-Tokushima fatty rats (OLETF, diabetic) and 12 age-matched male Long-Evans-Tokushima rats (LETO, healthy) with 200 µg/day G-CSF or saline for 5 consecutive days. Body weight reduction was greater in G-CSF-treated OLETF (G-CSF/OLETF) than saline-treated OLETF (saline/OLETF) following 8 weeks of treatment (−6.9±1.6% vs. −3.1±2.2%, *p*<0.05). G-CSF treatment had no effect on body weight in LETO or on food intake in either OLETF or LETO. Body fat in G-CSF/OLETF was more reduced than in saline/OLETF (−32.2±3.1% vs. −20.8±6.2%, *p*<0.05). Energy expenditure was higher in G-CSF/OLETF from 4 weeks after the treatments than in saline/OLETF. Serum levels of cholesterol, triglyceride, interleukin-6 and tumor necrosis factor-α were lower in G-CSF/OLETF than in saline/OLETF. Uncoupling protein-1 (UCP-1) expression in brown adipose tissue (BAT) was higher in G-CSF/OLETF than in saline/OLETF, but was unaffected in LETO. Immunofluorescence staining and PCR results revealed that G-CSF receptors were expressed in BAT. In vitro experiments using brown adipocyte primary culture revealed that G-CSF enhanced UCP-1 expression from mature brown adipocytes via p38 mitogen-activated protein kinase pathway. In conclusion, G-CSF treatment reduced body weight and increased energy expenditure in a diabetic model, and enhanced UCP-1 expression and decreased inflammatory cytokine levels may be associated with the effects of G-CSF treatment.

## Introduction

Granulocyte-colony stimulating factor (G-CSF) is a cytokine found in vertebrates that primarily acts as a regulator in the formation and development of neutrophils [1]. G-CSF is also a member of the class-1 cytokine superfamily. Many members of this superfamily have anti-obesity properties that take effect through changing appetite, energy expenditure or adipose tissue development. Such cytokines include granulocyte monocyte colony-stimulating factor (GM-CSF), ciliary neurotrophic factor (CNTF), interleukin-6 (IL-6), leukemia inhibitory factor (LIF) and leptin [2]–[9]. The molecular structure and intracellular signaling pathway of G-CSF receptors (G-CSFR) are similar to those of leptin, CNTF, LIF and IL-6 receptors [2], [10]. Their intracellular signaling pathway commonly includes Janus kinase/signal transducer and activator of transcription (JAK/STAT) and mitogen-activated protein kinase (MAPK), which are also associated with appetite, adipose tissue development and energy homeostasis [2], [11]–[13]. Obesity accompanies chronic systemic inflammation, characterized by pro-inflammatory cytokines including IL-6 and tumor necrosis factor-α (TNFα), which are also known to accelerate insulin resistance and other metabolic abnormalities of obesity [14]–[16]. G-CSF blunts the activation of major inflammatory cytokines, including TNFα and attenuates the harmful responses of inflammatory reactions [17], [18]. Moreover, in our previous experiments on the effects of G-CSF on diabetic neuropathy, we found unexpected weight loss in Otsuka-Long-Evans-Tokushima fatty (OLETF) rats treated with G-CSF. For these reasons, we hypothesized that G-CSF might also play a role in regulating energy homeostasis and body weight.

Obesity is a disorder associated with an imbalance between food intake and energy expenditure. Appetite and energy expenditure are primarily governed by hypothalamic mediators such as neuropeptide Y (NPY), agouti-related peptide (AgRP), pro-opiomelanocortin (POMC) and cocaine-amphetamine regulated transcript (CART) [19]. Leptin and many other class-1 cytokines influence these hypothalamic mediators and reduce appetite or enhance energy expenditure [5], [8], [19]–[22]. The best-known mechanism of the hypothalamus to control energy expenditure via the autonomic nerve system is BAT non-shivering thermogenesis, in which uncoupling protein-1 (UCP-1) plays a key role [23]. Leptin and CNTF also enhance the thermogenesis of BAT [23], [24]. Therefore, we investigated whether an alteration in body weight occurred after G-CSF treatment and whether it resulted from alterations in food intake or energy expenditure. We also investigated the effects of G-CSF treatment on appetite mediator expressions in the hypothalamus and UCP-1 expression in BAT to gain additional insight into alterations of food intake or energy expenditure.

## Materials and Methods

### Animals

This study was performed according to the ARRIVE guidelines for animal research [25]. The Hanyang University Institutional Animal Care and Use Committee approved all protocols. Male OLETF rats, characterized by hyperphagia due to a mutated cholecystokinin-1 receptor that results in an obese diabetic phenotype, were used as the animal model for obesity. Male Long-Evans Tokushima (LETO) rats were used as healthy non-diabetic controls of similar body size. The rats were supplied by the Tokushima Research Institute, Otsuka Pharmaceutical (Tokushima, Japan). OLETF rats are well-established animal models of obesity [26] and type 2 diabetes mellitus [27]. The animals were maintained in the Hanyang University Medical School Animal Experiment Center in a specific, pathogen-free facility at a controlled temperature (23±2°C) and humidity (55±5%) with a 12-hour artificial light and dark cycle. All animals were provided *ad libitum* with standard rodent chow (20.14% protein, 13.12% moisture, 5.9% fat and 5.02% fiber; Lab Rodent Chow; 38057; Purina Korea Inc., Seoul, South Korea).

### Animal models and experimental design

**Figure 1:**
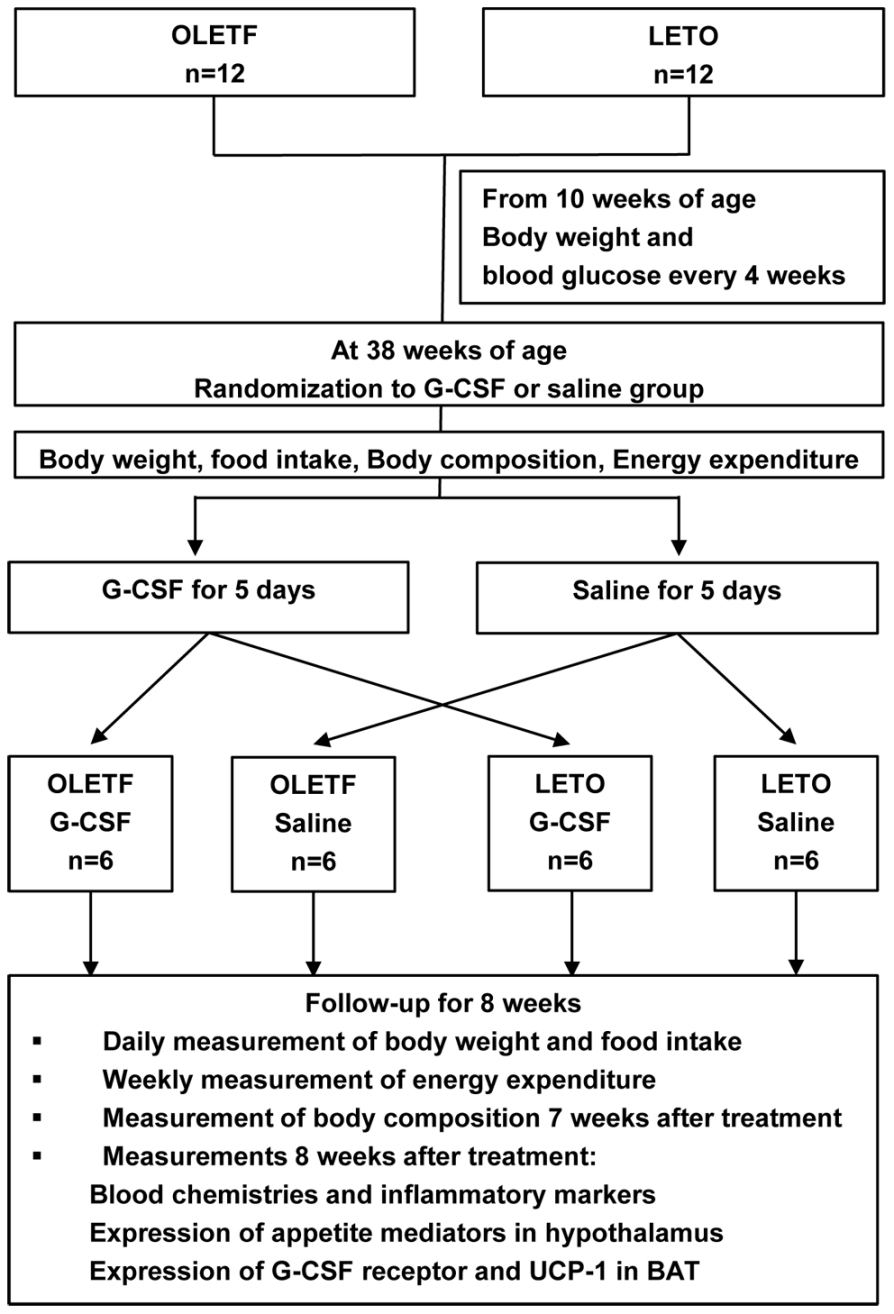
Schematic description of the experimental protocol. Twelve OLETF and 12 LETO rats were prepared at 10 weeks of age and randomized into either a G-CSF or saline treatment group. All animals were treated with G-CSF or saline for 5 consecutive days and were followed for 8 weeks. Body composition was measured using dual energy X-ray absorptiometry, and energy expenditure was measured with an indirect calorimeter. OLETF, Otsuka Long-Evans Tokushima Fatty rats; LETO, Long-Evans Tokushima Otsuka rats; G-CSF, granulocyte-colony stimulating factor; UCP-1 uncoupling protein-1; BAT, brown adipose tissue.

The experimental protocol is shown schematically in Figure 1. We obtained 12 OLETF and 12 LETO rats at 4 weeks of age and measured body weight and blood glucose every 4 weeks from 10 weeks of age to 38 weeks of age. At 38 weeks, when the body weights of the OLETF and LETO rats approached a similar point, the OLETF and LETO groups were each randomly divided into two subgroups: G-CSF-treated OLETF rats (G-CSF/OLETF), saline-treated OLETF rats (saline/OLETF), G-CSF-treated LETO rats (G-CSF/LETO) and saline-treated LETO rats (saline/LETO). We then administered 200 µg/kg of human recombinant G-CSF (Leucostim; Dong-A Pharmaceutical, South Korea) intra-peritoneally for 5 days to the G-CSF-treated groups. The same volume of saline was administered to the saline-treated groups in the same manner. All animals were followed for 8 weeks after treatment. Body weight and food intake were measured daily. Energy expenditure was measured with an indirect calorimeter at the end of the week before the treatments and at the end of every week for 7 weeks after the treatments. Dual energy X-ray absorptiometry (DEXA) was measured in all animals at the week before treatment and 7 weeks after treatment. The animals were euthanized 8 weeks after the treatments and the expression of UCP-1 in BAT and of the neurotransmitters for appetite in the hypothalamus were analyzed. Blood samples for biochemical analyses were taken just before the animals were euthanized.

### Analysis of biochemical profiles and inflammatory markers

Blood samples were taken from the tail vein after 8 hours of fasting. Sera were obtained by centrifugation and stored at −70°C. Serum glucose, total cholesterol, triglyceride and free fatty acid (FFA) levels were measured using an Olympus AU400 auto analyzer (Olympus GmbH, Germany). Insulin, IL-1*β*, IL-6 and TNFα were measured using a Milliplex Analyzer Luminex 200 System (Millipore, USA) with a Rat Cardiovascular Disease Panel kit.

### Body composition

The lean body and fat composition of the animals was measured by DEXA using a Hologic QDR 4500 device (Hologic Inc., USA) with an internal adapted collimator for small animal measurements (Hologic QDR Software for Windows XP version). The scan field was adjustable to a maximum of 36 cm×18 cm and spatial resolution was approximately 1 mm. All animals were anesthetized with Ketalar® (75 mg/kg) and Rompun® (5–10 mg/kg) before the measurement. After anesthesia, the animals were positioned ventrally on a reference film with all limbs extended.

### Energy expenditure

VO_2_ and VCO_2_ were measured in all animals for 6 hours at the end of every week using an indirect calorimeter (Oxymax; Columbus Instruments, USA). Energy expenditure was calculated from the following formula provided by the manufacturer:

Energy expenditure (kcal) = 3.815+1.232×(VCO_2_/VO_2_)×VO_2._

The results were transformed into values for 24 hours and normalized by total body weight (kg).

### Brown adipocyte isolation, culture and differentiation

Brown preadipocytes were obtained from the interscapular BAT of postnatal (1–2 days after birth) Sprague-Dawley rats. Isolation and culture of brown adipocytes were performed, as described previously [28]. The obtained tissues were dispersed in isolation buffer containing 100 µg collagenase (Sigma-Aldrich, South Korea) and 4% bovine serum albumin (BSA) (GenDEPOT, USA). The isolation buffer was composed of 0.123 M NaCl, 5 mM KCl, 1.3 mM CaCl2, 5 mM glucose and 100 mM hydroxyethyl piperazineethanesulfonic acid (all Sigma-Aldrich, South Korea), and maintained at 4°C. After 45 minute incubation with shaking at 37°C, isolated cells were suspended briefly in a 1∶3 mixture of erythrocyte lysis buffer (Lonza, USA) and maintenance media (Delbecco’s Modified Eagle’s Medium [DMEM] containing 1% penicillin and 20% fetal bovine serum [FBS] [all Sigma-Aldrich, South Korea]). After centrifuged at 1000 rpm for 5 minutes, isolated cells were cultured in the maintenance media at 37°C in a humidified atmosphere with 5% CO_2_.

Brown preadipocytes were induced to differentiate to mature brown adipocytes 2 days after confluence (day 0), as described previously [29]. The cells were briefly exposed to media containing DMEM with 10% FBS and a differentiation cocktail (0.5 mM isobutylmethylxanthine, 0.5 µM dexamethasone, 20 nM insulin, 125 µM indomethacin and 1 nM 3,3′,5-triiodo-L-thyronine [T3] [Sigma-Aldrich, South Korea]) and then placed in a maintenance medium composed of DMEM, 10% FBS, 1 nM T3 and 20 nM insulin. Maintenance media was replenished every 2 days.

### Oil red-O staining for cultured brown adipocytes

Oil red-O (Sigma-Aldrich, South Korea) staining was used to identify the lipid droplets of mature brown adipocytes on day 0 and 4 days after differentiation (day 4), as described previously [30].

### Immunofluorescence stain for G-CSFR

Immunofluorescence staining for G-CSFR was performed to locate G-CSFR in BAT and on the surface of brown adipocytes. The interscapular BAT was obtained from a 20 week-old male OLETF rat. The BAT sections were blocked with 0.1% BSA containing 10% normal goat serum (NGS) (all Sigma-Aldrich, South Korea) for 1 hour, and were incubated for 90 minutes with mouse monoclonal anti-G-CSFR primary antibodies (1∶100 dilution; Santa Cruz Biotechnology Inc., USA). The sections were washed and then incubated with fluorescein isothiocyanate-conjugated secondary antibody (1∶500 dilution; Abcam, USA) for 60 minutes. Brown adipocytes were fixed with 4% paraformaldehyde. After washed, the cells were exposed to 0.3% TritonX-100 for 10 minutes at room temperature to be permeable. Then, the cells were blocked with 0.1% BSA containing 10% NGS (all Sigma-Aldrich, South Korea) for 1 hour. The cell were then, incubated for 2 hours with a monoclonal anti-G-CSFR antibody as the primary antibody (1∶50 dilution; Santa Cruz Biotechnology Inc., USA). After washed again, the cells were incubated with fluorescein isothiocyanate-conjugated secondary antibody (1∶500; Abcam, USA) for 60 minutes. Images were obtained on an ECLIPSE 80i microscope equipped with an iAi progressive scan camera (Nikon, Tokyo, Japan) and CytoVision system software (Applied Imaging, UK).

### G-CSF and p38 MAPK inhibitor treatment on brown adipocytes

Mature brown adipocytes were exposed to a maintenance media containing G-CSF for 30 minutes on day 4. The optimal G-CSF concentration was determined over multiple attempts by elevating the G-CSF concentration in log scale. In some experiments, mature brown adipocytes were pretreated with 10 µM p38 MAPK inhibitor (SB203580; Santa Cruz Biotechnology, USA) for 1 hour before G-CSF treatment.

### PCR for appetite mediator, G-CSFR, brown adipocyte differentiation marker and UCP-1

Hypothalami were obtained to perform quantitative PCR (Q-PCR) for NPY, POMC, CART and AgRP, RT-PCR for G-CSFR and Q-PCR for UCP-1 were performed in BAT, and cultured brown adipocytes were obtained on day 0 and day 4 for RT-PCR of G-CSFR, UCP-1, PR domain containing 16 (PRDM16), peroxisome proliferator-activated receptor-γ (PPARγ) and PPAR-γ co-activator 1-α (PGC-1α). RNA was extracted using Qiazol reagent (Qiagen, USA). The primers used in the analyses are shown in Table 1.

**Table 1:** Primers for PCR.

Primer	Sequences (5′ to 3′)		Size (bp)
RT-PCR			
UCP-1	Forward	CCG GTG GAT GTG GTA AAA AC	242
	Reverse	CTC CAA GTC GCC TAT GTG GT	
PRDM16	Forward	ACC ATG CAC TTT TAG ATG AGA AGG A	526
	Reverse	ATC ATT GCA TAT GCC TGG TTC TTA G	
PGC-1α	Forward	CCT TAT TTT CTC AAA GAC CCC AAA G	516
	Reverse	CGT CAC AGG TGT AAC GGT AGG TAA T	
PPARγ	Forward	ACT CCC ATT CCT TTG ACA TC	265
	Reverse	TCC CCA CAG ACT CGG CAC TC	
G-CSFR	Forward	CCA TTG TCC ATC TTG GGG ATC	234
	Reverse	CCT GGA AGC TGT TGT TCC ATG	
GAPDH	Forward	GAC AAC TTT GGC ATC GTG GA	133
	Reverse	ATG CAG GGA TGA TGT TCT GG	
Q-PCR			
UCP-1	Forward	GGG CTG ATT CCT TTT GGT CTC	68
	Reverse	GGG TTG CAC TTC GGA AGT TGT	
POMC	Forward	CCA GGC AAC GGA GAT GAA	426
	Reverse	CTT GTC CTT GGG CGG GTT	
CART	Forward	ACG CAT TCC GAT CTA TGA	424
	Reverse	AAG CGA AAG TCC CTC TTC	
NPY	Forward	GTG GAC TGA CCC TCG CTC TA	231
	Reverse	GGG CAT TTT CTG TGC TTT CT	
AgRP	Forward	GCA GAC CGA GCA GAA CAT	129
	Reverse	CTG TTG TCC CAA GCA GGA	
GAPDH	Forward	CCT TCT CTT GTG ACA AAG TGG ACA T	96
	Reverse	CGT GGG TAG AGT CAT ACT GGA ACA T	

UCP-1, uncoupling protein-1; PRDM16, PR domain containing 16; PPARγ Peroxisome proliferator-activated receptor γ; PGC-1α, PPAR γ co-activator 1-α; G-CSFR, granulocyte-colony stimulating factor; POMC, pro-opiomelanocortin; CART, cocaine amphetamine regulated transcript; NPY, neuropeptide Y; AgRP, agouti-related peptide; GAPDH, glyceraldehyde 3-phosphate dehydrogenase.

#### RT-PCR for G-CSFR, UCP-1, PRDM16, PPAR and PGC-1α

RNA samples (1 µg) were reverse-transcribed with Moloney murine leukemia virus reverse transcriptase (Invitrogen Co, USA). PCR mixtures contained 1 µl cDNA, 2 µl 10X PCR buffer, 2 µl dNTP, 0.2 µL Taq DNA polymerase (iNtRON, South Korea) and 0.4 µl 25 pmol/µl of sense and anti-sense primers (Bioneer, South Korea). We performed PCR using a C1000TM Thermal Cycler (Bio-Rad, USA) under the following conditions: initial denaturation at 95°C for 5 minutes, 30 amplification cycles (denaturation at 95°C for 30 sec, annealing at 60°C for 30 sec, extension at 72°C for 1 minutes) and final extension at 72°C for 5 minutes. The products were visualized on 2% agarose gels stained with ethidium bromide using Gel-Doc 2000 (Bio-Rad, USA). For quantification of UCP-1 expression levels in brown adipocytes, gels were scanned, and pixel intensity for each band was determined using the Image J software (NIH Image, USA) and normalized to the intensity of GAPDH.

#### Q-PCR for NPY, POMC, CART, AgRP and UCP-1

RNA samples (3 µg) were reverse-transcribed as above. Expression was quantified using a Light Cycler® 480 System (Roche, Switzerland) with a FastStart DNA Master SYBR Green I kit (Roche Diagnostics, USA). We performed PCR using the following steps: incubation for 10 minutes at 95°C followed by 45 cycles of 10 sec at 95°C, 10 sec at 60°C and 8 s at 72°C and a final dissociation curve step at 65°C for 15 sec. The crossing point of each PCR was automatically determined by the LightCycler® program. PCR for all samples was run in duplicate. The transcript levels were normalized against those of GAPDH.

### Western blotting for UCP-1 and p38 MAPK activity

BAT was obtained from all of the animals for Western blotting for UCP-1. Mature brown adipocytes on day 4 were used to assess p38 MAPK activity by Western blotting. The samples were homogenized in ice-cold homogenization buffer (Pro-preb; iNtRON, South Korea). We transferred 50 µg proteins into a sample buffer that was then separated by 10% sodium dodecyl sulfate polyacrylamide gel electrophoresis and transferred to nitrocellulose membranes (0.45 µm pore size, Immobion-P; Bio-Rad, USA). After blocked in 5% skim milk solution for 60 minutes, the membrane was incubated with primary antibody for UCP-1 (1∶500; Calbiochem, USA), followed by incubation with HRP-conjugated anti-rabbit antibody (1∶2000; Jackson Immunoresearch, USA), as the secondary antibody. For p38 MAPK, we transferred 80 µg proteins. After the cells were blocked with 5% BSA for 60 minutes, the membrane was incubated with primary antibody for phosphorylated and total p38 MAPK (1∶1000; Cell Signaling Technology, USA), followed by incubation with the secondary antibody. GAPDH was used as a protein loading control. Positive protein bands were visualized using an ECL kit (Amersham Pharmacia Biotech, Piscataway, NJ, USA). The results were quantified with an image analyzer (Image lab 3.0, Bio-Rad, Hercules, CA, USA).

### Statistical analysis

The results are presented as the mean ± standard error of the mean. Mann-Whitney U-tests were performed to compare the changes in body weights and food intakes of the G-CSF-treated groups and the saline-treated groups each week after the treatments and to compare the changes in body composition, biochemical measurements and UCP-1 expression of the G-CSF-treated groups and the saline-treated groups. Kruskal-Wallis tests were used to compare body weights pre-treatment and at 8 weeks after G-CSF or saline treatment and the expression of hypothalamic appetite mediators in all 4 groups. A *p-*value <0.05 was considered statistically significant. All statistical analyses were performed using SPSS statistics 20.0 for Windows (IBM, New York, USA).

## Results

### Body weight and food intake changes

**Figure 2:**
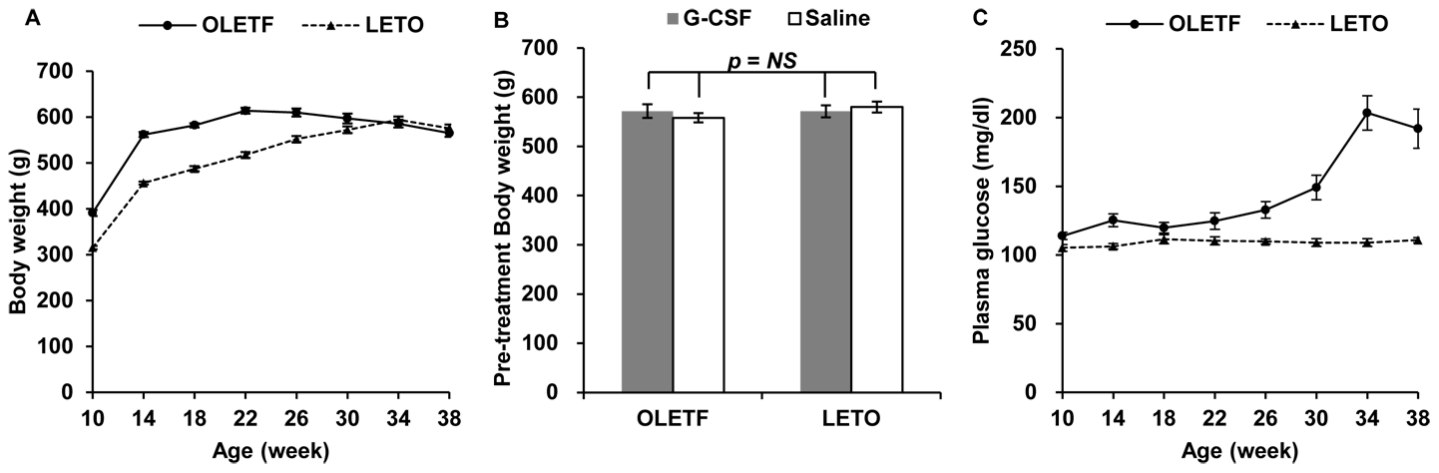
Body weight and blood glucose levels before G-CSF or saline treatment. (A) Body weights of the OLETF rats peaked at 22 weeks of age, then gradually decreased and were not significantly different with those of the LETO rats around 34 weeks of age; (B) Pre-treatment body weights at 38 weeks of age were not significantly different in all animal groups; (C) Blood glucose levels after 8 hours of fasting indicate the OLETF rats showed an overt diabetic phenotype after 30 weeks of age. - The data was presented as the mean ± S.E.M. OLETF, Otsuka Long-Evans Tokushima Fatty rats; LETO, Long-Evans Tokushima Otsuka rats; NS, not significant.

**Figure 3:**
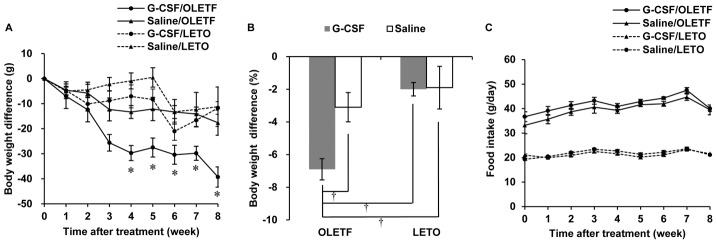
Changes in body weight and food intake after G-CSF or saline treatment. (A) Body weight difference from the pre-treatment body weight. Gradual decrease in body weight was shown in all animal groups, but the body weight reduction was significantly larger in G-CSF/OLETF than those in Saline/OLETF. The body weight reduction was not significantly different between G-CSF/LETO and Saline/LETO; (B) The percentage of body weight reduction in 8 weeks after the treatments was higher in G-CSF/OLETF than those in the other animal group; (C) The food intake was not significantly different between the G-CSF-treated groups and saline-treated groups of LETO and OLETF. * p<0.05 vs. saline/OLETF; † p<0.05 vs. other groups; - The data are shown as the mean ± S.E.M. G-CSF/OLETF, G-CSF-treated OLEFT; Saline/OLETF, saline-treated OLETF; G-CSF/LETO, G-CSF-treated LETO; Saline/LETO, saline-treated LETO; G-CSF, granulocyte colony-stimulating factor; OLETF, Otsuka Long-Evans Tokushima Fatty rats; LETO, Long-Evans Tokushima Otsuka rats.

The body weight of the OLETF rats peaked at 22 weeks of age, then gradually decreased and reached a similar value to that of the LETO rats at 34 weeks of age. The body weight of the LETO rats peaked at 34 weeks of age and then decreased at a rate similar to that of the OLETF rats (Figure 2A, Table S1). Pre-treatment body weights were not significantly different in the 4 groups (Figure 2B). Plasma glucose increased from 26 weeks of age and reached a diabetic level at 30 weeks of age in the OLETF rats but did not change in the LETO rats (Figure 2C, Table S2). During the 8 weeks after treatment with G-CSF or saline, body weights gradually decreased in both OLETF and LETO rats; however, greater body weight reductions were observed in the G-CSF/OLETF than in the saline/OLETF 4 weeks after treatment. On the contrary, body weight reductions were not significantly different between the G-CSF/LETO and saline/LETO groups in any week after treatment. The body weight reductions in the saline/OLETF were similar with those in the LETO rats (Figures 3A, Table S3). The percentage of body weight reduction from the pre-treatment body weight 8 weeks after treatment was higher in the G-CSF/OLETF than in the other groups, but was not significantly different in the G-CSF/OLETF, saline/LETO and G-CSF/LETO (Figures 3B). Food intakes in the OLETF rats were approximately twice those of the LETO rats; however, food intakes in all 4 groups were unaffected by treatment with G-CSF or saline (Figures 3C, Table S4).

### Body composition changes

**Figure 4:**
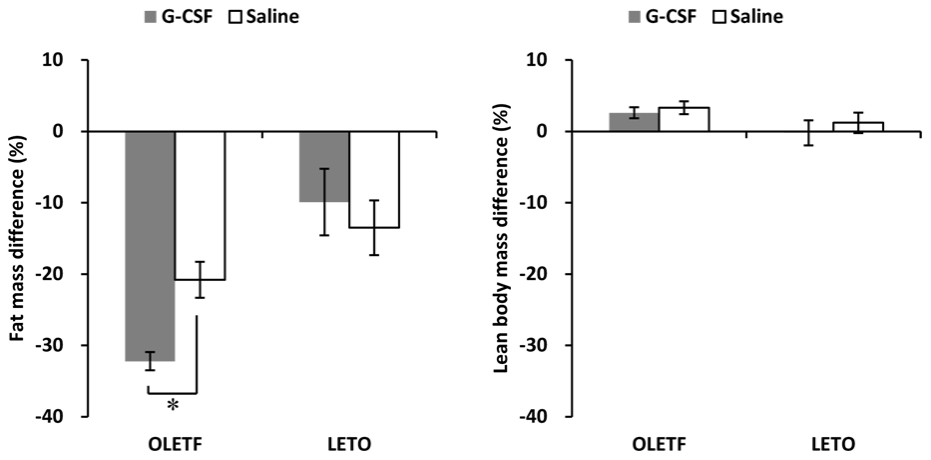
Changes in body composition. The fat mass reduction was significantly larger in G-CSF-treated OLETF than that in saline-treated OLETF, while was not different between G-CSF-treated LETO and saline-treated LETO. The lean body mass differences were not significantly different in all animal groups. * p<0.05; - The data were shown as the mean ± S.E.M. - Body composition was measured 1 week before (pre-treatment) and 7 weeks after (post-treatment) G-CSF or saline treatment. - Percentage differences are shown as the difference between the pre-treatment value and post-treatment value divided by the pre-treatment value. G-CSF, granulocyte colony-stimulating factor; OLETF, Otsuka Long-Evans Tokushima Fatty rats; LETO, Long-Evans Tokushima Otsuka rats.

The differences in the percentage of body fat mass in the G-CSF/OLETF were greater than those in the saline/OLETF. In contrast, the differences in lean body mass percentages were not significantly different between the G-CSF-treated groups and the saline-treated groups in both OLETF and LETO rats (Figure 4, Table S5).

### Energy expenditure changes

**Figure 5:**
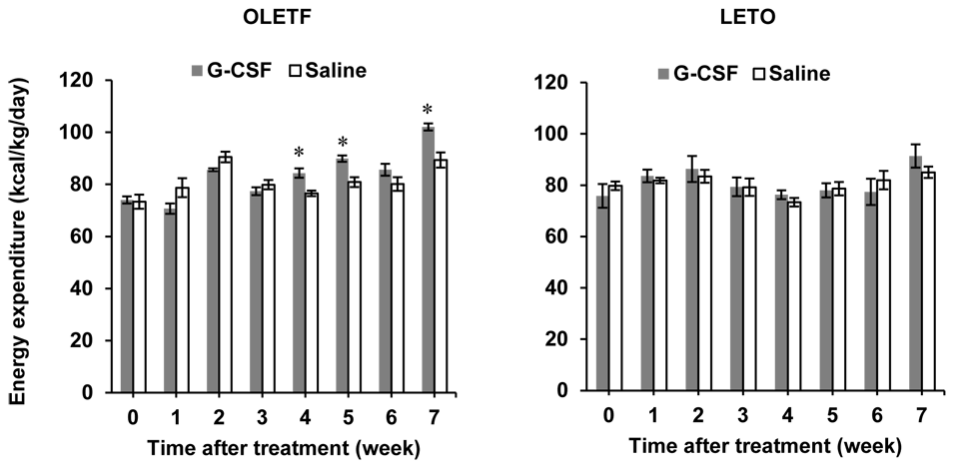
Energy expenditure after G-CSF and saline treatment. Energy expenditure in the G-CSF-treated OLETF rats was greater than in the saline-treated OLETF rats at 4, 5, and 7 weeks after treatment. Energy expenditure in the G-CSF-treated LETO was not different than that in the saline-treated LETO up to 7 weeks after the treatments. * p<0.05 vs. the saline-treated group. - The data were shown as the mean ± S.E.M. - Energy expenditure was measured with an indirect calorimeter. G-CSF, granulocyte colony-stimulating factor; OLETF, Otsuka Long-Evans Tokushima Fatty rats; LETO, Long-Evans Tokushima Otsuka rats.

Energy expenditures in the OLETF rats did not change significantly for 3 weeks after the treatments. However, 4, 5 and 7 weeks after the treatments, energy expenditures were significantly higher in the G-CSF/OLETF than in the saline/OLETF. Energy expenditures in the G-CSF/LETO and saline/LETO were not different during the experimental period (Figure 5, Table S6).

### Differences in biochemical and inflammatory markers

**Figure 6:**
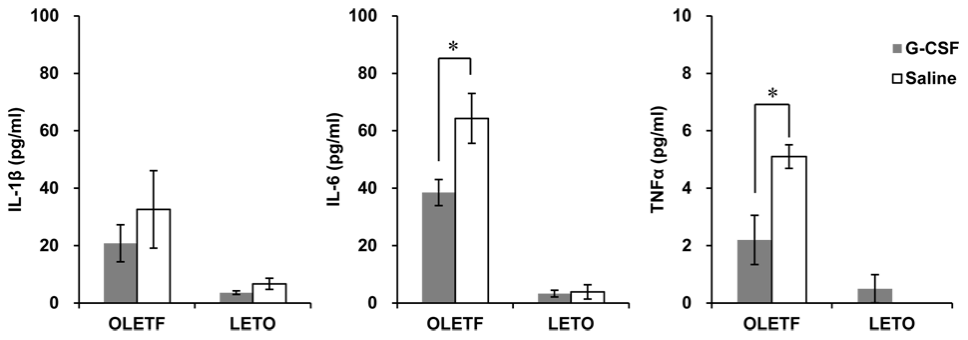
Serum inflammatory cytokines after treatment with G-CSF or saline. The IL-1β, IL-6 and TNFα levels were higher in OLETF than those in LETO in both treatments group. Serum levels of IL-6 and TNFα in the G-CSF-treated OLETF rats are significantly higher than in the saline-treated OLETF rats. There is no significant difference between serum levels of the cytokines in the G-CSF-treated group and those in the saline-treated group of LETO rats. * p<0.05; - The data were presented as the mean ± S.E.M. IL-1β, interleukin-1β; IL-6, interleukin-6; TNFα, tumor necrosis factor α; G-CSF, granulocyte colony-stimulating factor; OLETF, Otsuka Long-Evans Tokushima Fatty rats; LETO, Long-Evans Tokushima Otsuka rats.

Biochemical and inflammatory markers of the G-CSF-treated group and the saline-treated group of both OLETF and LETO rats were compared at 8 weeks after treatment. Serum levels of total cholesterol and triglyceride in the G-CSF/OLETF were lower than the saline/OLETF, whereas serum levels of glucose, insulin and free fatty acids were not significantly different in the G-CSF/OLETF and saline/OLETF. None of the biochemical test results were different in the G-CSF/LETO and saline/LETO rats (Table 2). Serum levels of IL-1β, IL-6 and TNFα were higher in the saline/OLETF than those in the saline/LETO (IL-1β 32.6±13.5 pg/ml vs. 6.7±2.0 pg/ml, *p* = 0.037; IL-6 64.3±8.7 pg/ml vs. 3.9±2.5 pg/ml, *p* = 0.003; TNFα 5.1±1.0 pg/ml vs. 0.0±0.0 pg/ml, *p* = 0.002). Serum levels of IL-6 and TNF*α* in the G-CSF/OLETF rats were lower than those in the saline/OLETF, whereas serum levels of IL-1*β* were not different in the G-CSF/OLETF and saline/OLETF. There was no difference between serum levels of the inflammatory cytokines in the G-CSF/LETO and those in the saline/LETO (Figure 6, Table S7).

**Table 2:** Biochemical test results after treatment with G-CSF or saline.

	OLETF		LETO	
	G-CSF	Saline	G-CSF	Saline
Glucose (mg/dl)	208.8±20.6	198.5±21.8	109.5±2.4	107.8±2.5
Total cholesterol (mg/dl)	212.5±7.5*	254.8±10.9	110.3±2.9	115.2±6.9
Triglyceride (mg/dl)	102.3±27.6*	161.7±39.4	36.0±9.4	32.3±1.8
Free fatty acid (µEq/l)	675.8±38.1	698.5±24.7	577.5±46.4	554.7±31.8
Insulin (mIU/l)	12.0±0.6	13.6±1.3	12.7±3.3	10.3±2.3
HOMA-IR	6.2±2.0	6.6±0.6	3.4±0.8	2.8±0.7

* *p*<0.05 compared to saline-treated groups.

- Data was shown as the mean ± S.E.M.

G-CSF; granulocyte-colony stimulating factor; OLETF, Otsuka Long-Evans Tokushima Fatty rats; LETO, Long-Evans Tokushima rats.

### mRNA expression of hypothalamic appetite mediators

**Figure 7:**
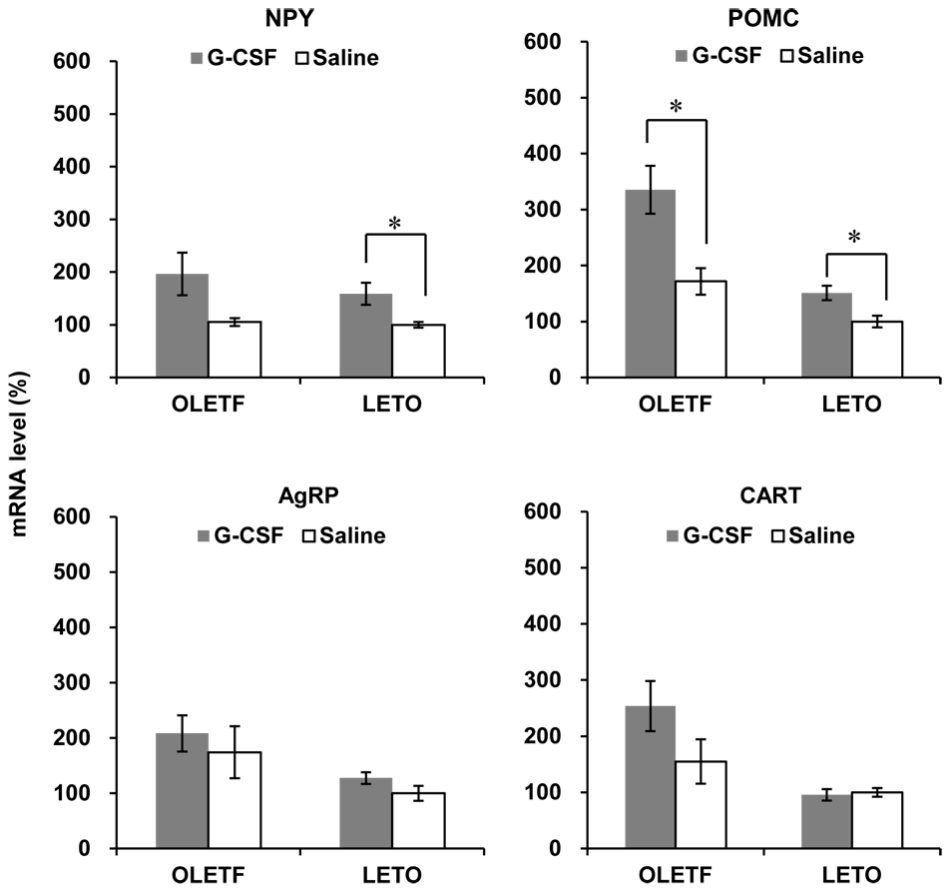
Hypothalamic expression of appetite mediators. The mRNA levels of NPY were not different between the two groups of OLETF, while significantly higher in G-CSF-treated LETO than those in saline-treated LETO. The mRNA levels of POMC were significantly higher in G-CSF-treated groups than those in saline-treated groups in both OLETF and LETO. The mRNA levels of AgRP and CART were not different between G-CSF-treated groups and saline-treated groups in both OLETF and LETO. * p<0.05. - The data were presented as the mean ± S.E.M. - The hypothalamic expression of POMC in the G-CSF-treated OLETF. - All measurements were duplicated, and the mRNA levels were normalized against those of the saline-treated LETO rats. NPY, neuropeptide Y; POMC, pro-opiomelanocortin; AgRP, agouti-related peptide; CART, cocaine-amphetamine regulated transcript; G-CSF, granulocyte colony-stimulating factor; OLETF, Otsuka Long-Evans Tokushima Fatty rats; LETO, Long-Evans Tokushima Otsuka rats.

Hypothalamic NPY mRNA levels were not different between the two OLETF groups, but were higher in the G-CSF/LETO than those in the saline/LETO. The mRNA levels of POMC were higher in the G-CSF-treated groups than that in the saline-treated groups in both OLETF and LETO rats. There were no significant differences in hypothalamic mRNA levels of AgRP and CART between the two groups of OLETF and LETO rats (Figure 7, Table S8).

### Expression of UCP-1 and G-CSFR in BAT

**Figure 8:**
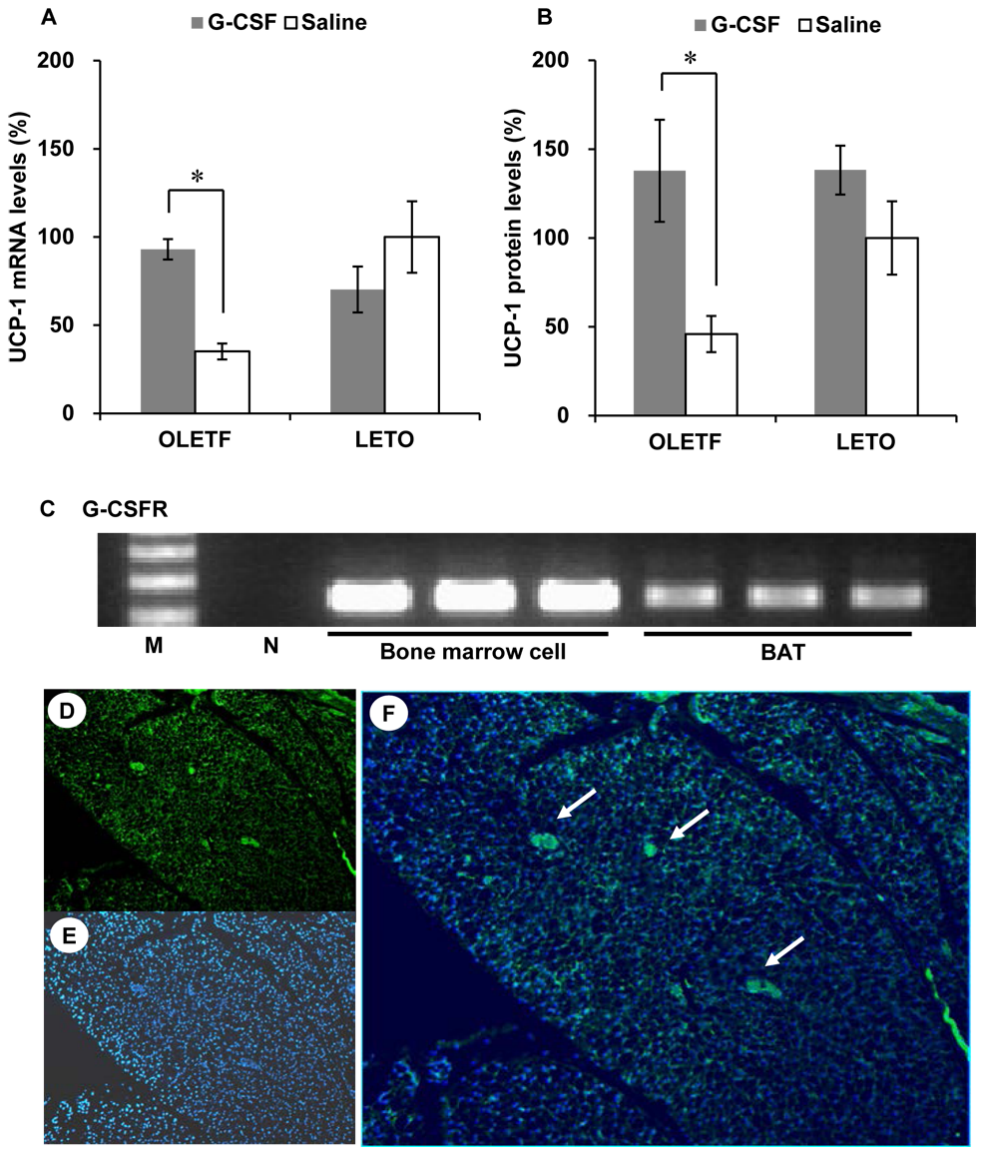
UCP-1 and G-CSFR expression in BAT. (A) UCP-1 mRNA levels are higher in the G-CSF-treated OLETF than those in the saline-treated OLETF. UCP-1 mRNA levels are unaffected by G-CSF treatment in LETO; (B) UCP-1 protein levels are higher in the G-CSF-treated OLETF than those in the saline-treated OLETF. UCP-1 protein levels are unaffected by G-CSF treatment in LETO; (C) RT-PCR results show the presence of G-CSFR in BAT; (D), (E) and (F) Immunofluorescence staining revealed that G-CSFR was presented in the islands of brown adipocytes (white arrows) (D, immunofluorescence staining for G-CSFR, 200; E, DAPI, 200; F, merged, 200). * p<0.05. - The data were presented as the mean ± S.E.M. - Measurements of UCP-1 mRNA levels were duplicated, and the mRNA levels were normalized against those of the saline-treated LETO rats. - UCP-1 protein levels were normalized against those of the saline-treated LETO rats. UCP-1, uncoupling protein-1; G-CSFR, G-CSF receptor; BAT, brown adipose tissue.

Values in the G-CSF/OLETF were higher than those of the saline/OLETF 8 weeks after the treatments; however, there was no difference between the UCP-1 mRNA levels in BAT in the G-CSF/LETO and those in saline/LETO. UCP-1 protein levels in the G-CSF/OLETF were also higher than those in the saline/OLETF rats, whereas no difference was found in the G-CSF/LETO and the saline/LETO (Figures 8A and B, Table S9). G-CSFR mRNA was expressed in BAT (Figure 8C). The immunofluorescence staining for G-CSFR revealed that G-CSFR was mainly present in the islands of brown adipocytes in the inter-scapular BAT of a 20 week-old male OLETF rat (Figure 8D, E and F).

### UCP-1 expression in brown adipocytes by G-CSF via p38 MAPK pathway

**Figure 9:**
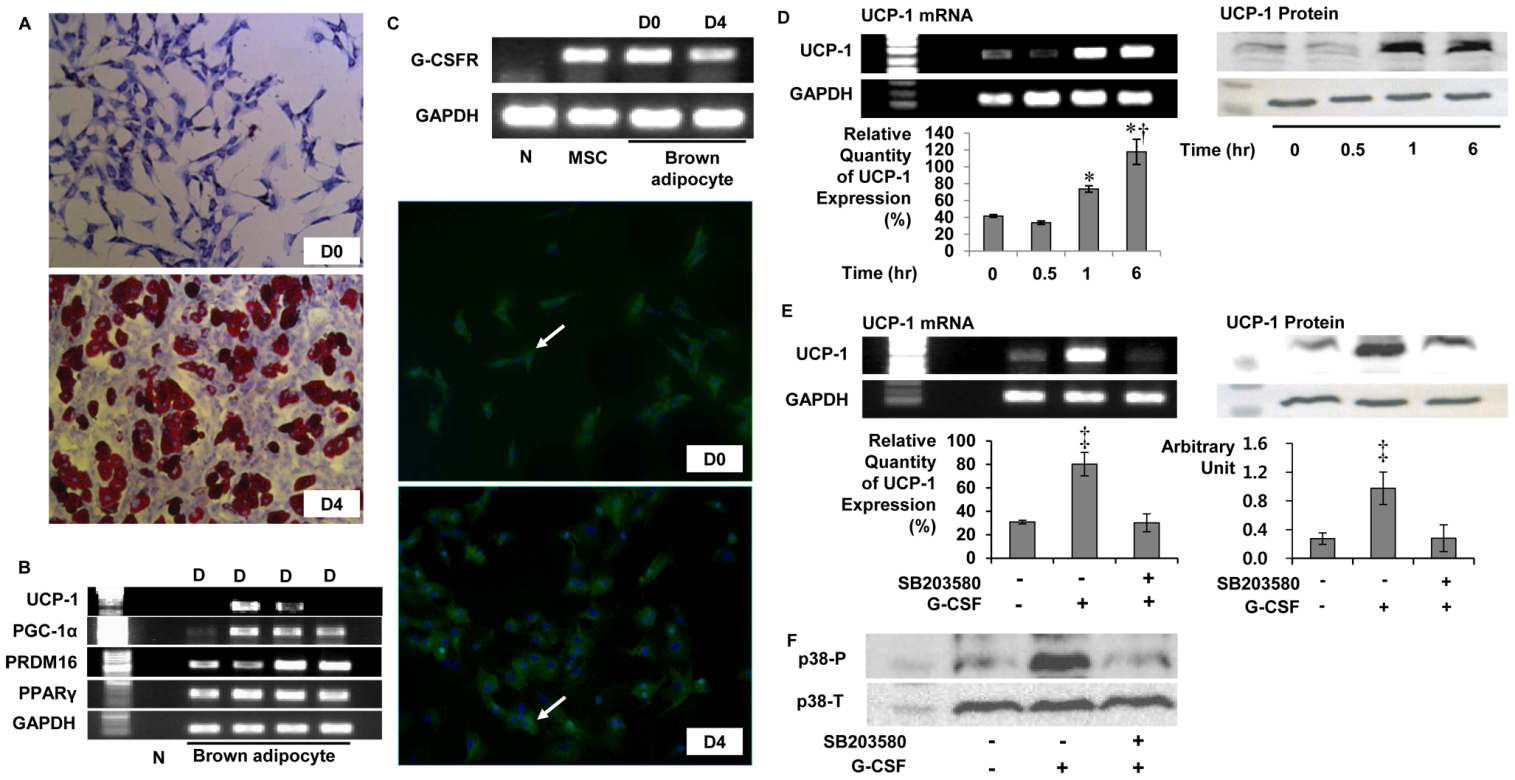
UCP-1 expression in primary cultured brown adipocytes by G-CSF via p38 MAPK pathway. (A) Oil red-O staining for brown adipocytes; (B) Indictors for differentiation of brown adipocytes. UCP-1 were expressed from D2 to D4 and the band density were decreased on D4; (C) mRNA expression and immunofluorescence staining of G-CSFR in primary cultured brown adipocytes. Cytoplasm was stained by G-CSFR specific antibodies (white arrow); (D) UCP-1 mRNA and protein expression in mature brown adipocytes on D4. After 1 hour from G-CSF treatment, UCP-1 expressions were significantly enhanced. (E) UCP-1 mRNA and protein expression was enhanced after G-CSF treatment and repressed after G-CSF treatment when brown adipocytes were pretreated with SB203580 (p38 MAPK inhibitor); (F) p38 MAPK phosphorylation was enhanced 30 minutes after G-CSF treatment and repressed by SB203580 pretreatment. * p<0.05 vs. UCP-1 level at 0 hour; † p<0.05 vs. UCP-1 levels at 1 hour; ‡ p<0.05 vs. the others. - The relative quantities of UCP-1 mRNA were measured 5 times and averaged. D(n), n days after differentiation; G-CSFR, Granulocyte colony-stimulating factor receptor; GAPDH, Glyceraldehyde 3-phosphate dehydrogenase; MSC; Bone marrow derived mesenchymal stem cell; UCP-1, Uncoupling protein-1; PPAR Peroxisome proliferator-activated receptor; PGC-1α, PPAR γ co-activator 1-α; SB203580; p38 Mitogen-activated protein kinase inhibitor; p38 MAPK, p38 Mitogen-activated protein kinase; p38-P, phosphorylated form of p38 MAPK; p38-T, total form of p38 MAPK.

Because G-CSF treatment increased energy expenditure and UCP-1 expression in BAT in OLETF and G-CSFRs were expressed in BAT, we performed additional experiments using brown adipocyte primary culture whether G-CSF could directly enhance UCP-1 expression in brown adipocytes. After exposure to differentiation cocktail, brown preadipocytes differentiated into mature brown adipocytes containing lipid droplets and expressed differentiation indicators including PRDM16, PGC-1α, PPARγ and UCP-1 2 days after differentiation. UCP-1 expression intensities were decreased at 4 days after differentiation (day 4) and not observed at 6 days after differentiation (Figure 9A and B). G-CSFR mRNA was expressed in brown preadipocytes (day 0) and in mature brown adipocytes (day 4), and immunofluorescence staining demonstrated that G-CSFRs were present on brown preadipocytes and mature brown adipocytes (Figure 9C). UCP-1 expression was observed at 1 hour and 6 hours after brown adipocytes were exposed to 3 µg/mL (6.5 µM) G-CSF. The relative quantities of UCP-1 expression mRNA also significantly increased after 1 hour from G-CSF treatment and the UCP-1 protein levels were also increased after 1 hour from G-CSF treatment (Figure 9D). However, UCP-1 mRNA and protein expressions were not enhanced after G-CSF treatment when brown adipocytes were pretreated with p38 MAPK inhibitor (SB203580; Sigma-Aldrich, USA) (Figure 9E). p38 MAPK phosphorylation which was enhanced after G-CSF treatment, was repressed after G-CSF treatment, when the brown adipocytes were pretreated with SB203580 (Figure 9F).

## Discussion

G-CSF is a member of the class-1 cytokine superfamily, many other members of which are known to play a role in energy homeostasis, and its intracellular signal pathways are similar to many cytokines that have known anti-obesity effects [1], [2], [11]. For example, IL-6, CNTF, LIF, GM-CSF and leptin are known to alter appetite, glucose and lipid metabolism, and energy expenditure [3]–[9]. We found, for the first time, that G-CSF treatment reduced body weight without altering appetite in OLETF rats. Unlike many inflammatory cytokines (such as IL-1 and IL-6) that have been identified as causing cachectic effects [31], the effect of G-CSF results not from cachectic effects, but from anti-obesity effects. Body weight reduction after G-CSF treatment was mainly due to a reduction in fat components. The muscle mass represented by the lean body components was not affected by G-CSF treatment. The body weight reduction was accompanied by some beneficial effects, including lower blood levels of total cholesterol and triglyceride and reduced levels of cytokines closely associated with fat inflammation, such as TNFα and IL-6 [14]–[16]. Increases in energy expenditure and UCP-1 expression in BAT were observed after G-CSF treatment in OLETF rats. It was also first found that brown preadipocytes, mature brown adipocytes and BAT expressed G-CAFR and that G-CSF enhanced UCP-1 expression in brown adipocytes via p38 MAPK pathway. This could be one of mechanisms to explain increased energy expenditure and UCP-1 expression in OLETF rats after G-CSF treatment.

It has been reported that the body weights of OLETF rats peak approximately 30 weeks of age and then gradually decrease [32]. In our data, the body weights of OLETF rats began to decrease at 26 weeks of age. Decreasing body weights of the OLETF rats at the time of treatment may cause concerns about the effects of G-CSF on the body weights of OLETF rats. We started the G-CSF and saline treatment at 38 weeks of age, because the body weights of both animals had approached at a similar level at 34 weeks of age and then decreased at a similar rate. The blood glucose levels in the OLETF rats also reached overt diabetic levels after 34 weeks of age. Because we had observed previously that G-CSF treatment does not affect the body weight of LETO rats, the healthy animal model without diabetes, it was important to start the treatments after the OLETF rats developed overt diabetes and to compare the effect of G-CSF on body weight in OLETF rats with that in LETO rats. The results show that the body weight reduction in G-CSF/OLETF was greater than that in saline/OLETF 4 weeks after the treatments, and the rates of body weight reduction were similar among the other animal groups.

The effect of G-CSF treatment was not observed in the LETO rats. Appetites were not affected by G-CSF treatment in the OLETF or LETO rats; however, the reductions of body weight and fat mass, the decreases in serum levels of cholesterol and triglyceride, the improvement in systemic inflammation and increases in energy expenditure and UCP-1 expression all appeared only in the G-CSF/OLETF rats. Although the body weights of both the OLETF and LETO rats were similar at the time of the treatments, the OLETF rats are a spontaneous animal model for type 2 diabetes with obesity, while the LETO rats are healthy controls. Therefore, the effects of G-CSF appear to be associated with metabolic and immunologic alterations that have occurred in a diabetic phenotype. The principle function of BAT is to regulate energy expenditure via non-shivering thermogenesis; UCP-1, a mitochondrial membrane protein, plays a key role [23]. UCP-1 expression in BAT is closely linked to obesity and diabetes [33]. Genetic ablation of BAT results in the development of obesity without hyperphagia in animal models [34]. UCP-1 polymorphism is also associated with the development of obesity and diabetes in many human studies [35]. In contrast, BAT thermogenesis and UCP-1 expression could also be affected by diabetes and insulin resistance conditions [23]. Diet-induced insulin resistance decreases BAT volume and hyperinsulinemia induces BAT atrophy [36], [37]. Hidaka et al. [38] reported that glucose uptake, fatty acid oxidation and UCP-1 expression in BAT were down-regulated in a streptozotocin-induced diabetic model and up-regulated in the same model when leptin was centrally infused. Therefore, we speculate that G-CSF treatment could restore UCP-1 expression in the diabetic model and might lead to increased energy consumption and weight reduction. Because fasting glucose and insulin levels did not change after G-CSF treatment, we also suggest that enhanced UCP-1 expression in BAT after G-CSF treatment is a process independent of insulin resistance.

The potential mechanisms for G-CSF treatment effects could be associated with enhanced UCP-1 expression in BAT. It is well-known that the hypothalamus regulates BAT non-shivering thermogenesis via sympathetic innervations [23]. Norepinephrine signals from the sympathetic nerve enhance thermogenesis by multiple pathways, including the stimulation of lipolysis to fuel thermogenesis, the enhancement of UCP-1 expression and the decrease in brown adipocytes apoptosis [23], [39]. Leptin stimulates sympathetic nerve signals to BAT via POMC expression in the hypothalamus [19], [23], Leptin receptors share many parts of intracellular domain structures and signaling pathways with G-CSFR [11], [12]. However, unlike leptin treatment, G-CSF treatment does not affect appetite or hypothalamic appetite mediators other than POMC in OLETF rats. And, hypothalamic POMC levels are higher in the G-CSF-treated rats of both OLETF and LETO groups. Moreover, no data for the geographical distribution of hypothalamic G-CSFR are included in our study. Therefore, it is difficult to suggest that increased POMC are associated with the effect of G-CSF treatment and enhanced UCP-1 expression in BAT. Further experiments are necessary to illuminate whether the increased POMC expression was due to the G-CSF treatments.

We demonstrated that the BAT of OLETF rats expressed G-CSFRs. The intracellular signaling pathway of G-CSF includes JAK/STAT and p38 MAPK [11]. A recent study showed that BNP stimulates UCP-1 expression in BAT via the p38 MAPK pathway [40]. Norepinephrine stimuli also activate p38 MAPK to increase UCP-1 expression in BAT [39]. CNTF has its receptors on BAT, which are structurally similar to G-CSFR and directly activates UCP-1 expression in BAT via p42/44 MAPK [24]. Our in vitro experiments also revealed that brown adipocytes express G-CSFRs and that G-CSF-treated mature brown adipocytes expressed UCP-1 mRNA via p38 MAPK activation. Therefore, enhanced UCP-1 expression in BAT could be associated with G-CSFR-mediated p38 MAPK activation.

Obesity is a low-grade systemic inflammatory condition mediated by cytokines such as TNFα and IL-6 [14]. TNFα has been reported to induce brown adipocytes apoptosis in primary culture, inhibit UCP-1 expression and mediate BAT atrophy in insulin resistance syndromes such as obesity and diabetes [37], [41], [42]. G-CSF attenuates the TNFα response to systemic inflammation [17], [18]. Similarly, we observed lower serum levels of IL-6 and TNFα in G-CSF/OLETF than in saline/OLETF. Therefore, this immune-modulatory effect of G-CSF might also have ameliorated BAT atrophy and contributed to body weight reductions in the diabetic model.

The major limitation of our study is that the detailed mechanisms of the body weight reduction effect of G-CSF were not sufficiently explored. In vitro experiments with brown adipocytes may partly resolve the question of the enhanced UCP-1 expression in BAT. However, the influence of the immune-modulatory function of G-CSF on BAT still requires further histological and molecular investigation. Long-lasting effects of G-CSF on body weight and energy expenditure are also difficult to explain from our data. G-CSF serum levels significantly decreased only several hours after intra-peritoneal G-CSF injection [43], it is difficult to suggest that the effects of G-CSF treatment are associated with metabolic effects of circulating G-CSF. However, we reported that diabetes-related conditions, diabetic nephropathy, steatohepatitis and diabetic cardiomyopathy were improved in OLETF rats 4 weeks after G-CSF treatment in the same manner [44]–[46], and the long-lasting effects of G-CSF in this study were also similar to those in our previous reports. Further investigations are necessary to determine the long-lasting effects of G-CSF. Genetic lack of cholecystokinin-1 receptor in OLETF rats induced the animal into a hyperphagic state [47]. The OLETF rat model is similar to the model of type 2 diabetes with mild obesity [48]. However, the lack of cholecystokinin receptors affects hypothalamic NPY and POMC expressions in OLETF rats [49]. Since UCP-1 expression is influenced by these hypothalamic appetite mediators, the effect of G-CSF on UCP-1 expression and energy expenditure could be different in other diabetic animal models. In addition, G-CSF action on the feeding behavior could be also affected in OLETF rats. Therefore, the effects of G-CSF on body weight should be also tested in other diabetic animal models.

In conclusion, G-CSF has anti-obesity effects in an animal model with diabetes and obesity. Treatment with G-CSF reduces body weight and fat components, improves lipid abnormalities and ameliorates systemic inflammation without altering appetite. The anti-obesity effect of G-CSF is not associated with improved insulin resistance or glycemic control but is associated with increased energy expenditure and enhanced UCP-1 expression in BAT in a diabetic animal model. The effects of G-CSF treatment on UCP-1 expression in BAT could be mediated by G-CSFR and p38 MAPK pathway or immune-modulatory function of G-CSF. Further experiments are needed to illuminate in detail the mechanisms of the effects of G-CSF on body weight reduction.

## Supporting Information

Table S1 Body weight before the treatments of G-CSF or saline.
(XLSX)

Table S2 Blood glucose level before the treatments of G-CSF or saline.
(XLSX)

Table S3 Body weight changes after the treatments of G-CSF and saline.
(XLSX)

Table S4 Weekly average food intakes after the treatments of G-CSF or saline.
(XLSX)

Table S5 Changes in body composition after the treatments of G-CSF and saline.
(XLSX)

Table S6 Energy expenditure changes after the treatment G-CSF or saline.
(XLSX)

Table S7 Serum inflammatory cytokines after the treatment of G-CSF and saline.
(XLSX)

Table S8 Quantitative PCR for appetite mediators in hypothalamus after the treatments of G-CSF and saline.
(XLSX)

Table S9 UCP-1 expression in brown adipose tissue after the treatments of G-CSF and saline.
(XLSX)
